# Annotations

**Published:** 1890-01-25

**Authors:** 


### Annotations.

**Lead Poisoning in Sheffield.**

We are indebted to the report by a "special commissioner" of the *Sheffield and Rotherham Independent* for an account of a remarkable outbreak of plumbism at Sheffield. The commissioner gives a table of 129 cases scattered over the town and district, and all certified by medical men of the place to be suffering or having suffered from lead poisoning within the last four years, and these cases were all not engaged in lead working. This is a most astonishing list. The cause has been found in the water, which as much as four years ago was proved by Dr. Sinclair White to contain a notable quantity of lead. *Dr. John Brown in a careful study of the disease as it occurred at Bacup during the last two years has found lead in proportion varying from one-tenth to two grains per gallon. The cause of lead getting into the drinking water has been stated to be due: First, to acidity of the water, this acidity being caused by sulphuric acid formed from the pyrites existing in the source of the water supply. Secondly, to the presence of organic germs, causing acidity. Dr. G. Kirker, in a note to the *British Medical Journal* of the 11th inst., states that the power of certain specimens of water to dissolve lead was directly proportional to the number of germs present. We venture to suggest that the amount of sulphur acids in the atmosphere which is in contact with the water may have also a small part in causing the acidity, which is potential in dissolving the lead. Where much coal and gas is burnt, there is always a considerable quantity of sulphur consumed, and this in the form of moist sulphurous acid, soon converted into sulphuric acid, can be easily absorbed by water stored in lead cisterns. We once tested the atmosphere at the top of a house where the ventilation was not good late at night, after much gas had been burnt at an entertainment. Amongst the products found were distinct traces of oxidised sulphur. Indoor cisterns are still to be found in many houses. At Sheffield, moreover, Dr. Sinclair White has pointed out that the acid in the water is sulphuric, and Dr. Brown, in his essay, states the same thing with regard to the water at Bacup. The methods recommended for removing lead from water so as to have it ready for use, are chiefly two: First, treating it with an alkaline carbonate as fresh limestone (Dr. White), or carbonate of soda (Dr. Brown) Secondly. By filtration. (*a*) By compound carbon filters. (*b*) By careful filtration at the ordinary filter beds of the water works, so arranging the process that it is continuous, and that the water gets thoroughly under the influence of the silica. According to the "commissioner's" report Sheffield is still in the same condition as it was in 1885, when Dr. White began his investigations. Though the works have been transferred from a private company to the Corporation, nothing has been done except to pass a resolution "that the Water Committee have requested their engineer (Mr. Eaton) to report on the effect of the Sheffield water on lead, and as to the best means of treating the water with a view of counteracting any such effect that it may have." "They decline the proposal for a special sub-committee to thoroughly investigate the matter, and apparently have yet to be convinced of what those best acquainted with the subject are unanimous in thinking should have been remedied long ago." If the above be really a true and fair representation of the facts, it is only another example of the spirit of Bumbledom which still resides in corporations, and also of the unfortunate results which sometimes ensue from converting private into public property. A private water company which dispenses contaminated water would in all probability be soon called to account, but when the owner of the water supply is a corporation, it is often a difficult task to bring the right person to book. Lead poisoning is an exceedingly dangerous thing, often ending in death and, what is almost as bad, causing a permanent state of ill-health. It appears to us that it is high time the citizens of Sheffield should stir up their Corporation and put a stop to the plague which is among them.

* "Clinical and Chemical Observations on Plumbism." By John Brown, M.D., etc., London. Simpkin Marshall and Co

**The Irish Lunacy Service.**

Judging from a leading article in the *Medical Press*, it would seem that the appointment of one of the visiting physicians of a district asylum to the post of resident superintendent has caused a good deal of unpleasant feeling in Irish lunacy circles, and has revived the old controversy as to the desirableness of having visiting physicians at all. We do not lay claim to any very intimate acquaintance with the Irish lunacy system, but we are so much opposed to anything which can have even the most remote relation to dual control that we take exception to the broad principle of appointing visiting physicians to district asylums. To us it seems self-evident that no medical duties can be attached to the post which could not be equally well done by the resident medical superintendent. Of course, it is now and then desirable in asylums, as well as out of them, to have a second medical opinion on a case, but this calling in of external aid should be left entirely in the hands of the resident medical superintendent, and the consultant should limit his duties to the case in question. If it be said that these visiting physicians are really inspectors, and are the representatives of the public, then let them be known as such, with proper rules and duties framed for them. It does not seem likely that Irish asylum medical officers can often have a slur cast on them by being passed over in favour of outsiders. The salaries attached to the posts are often under five or six hundred a year, and it cannot be supposed that any physician in practice would sacrifice his position and income for any such sum. Another thing is that the age of visiting physicians would preclude the possibility of their taking the appointments. The duties of a resident superintendent are so trying and irksome that, if conscientiously performed, they require that a man should be in the full vigour of life, and unless the attending circumstances are exceptional, his age should not exceed thirty-five on appointment. Our contemporary says that visiting physicians "are a necessary safeguard to the patients," while it tells us "of the existence of long continued abuses in the management" of the Limerick Asylum previous to the appointment of Dr. Courtenay. This is curious logic. If these officers are necessary safeguards, how came it that under the very system that the *Medical Press* is so much enamoured of grave abuses have been long continued? The admirable manner in which Dr. Courtenay managed the Limerick Asylum is well known in England, and if anyone thinks Dr. Courtenay's views on administration are wrong, let him contrast the condition of the Limerick Asylum before and after he assumed the reins of government. The whole article may be judged by one of the writer's concluding statements—namely, that the resident medical superintendents "are not capable of undertaking the treatment of the bodily diseases of the asylum inmates." This is really too much, for it amounts in effect to a declaration that the younger practitioners in Ireland do not understand even the rudiments of their profession.

